# Early-Warning Immune Predictors for Invasive Pulmonary Aspergillosis in Severe Patients With Severe Fever With Thrombocytopenia Syndrome

**DOI:** 10.3389/fimmu.2021.576640

**Published:** 2021-05-07

**Authors:** Lifen Hu, Qinxiang Kong, Chengcheng Yue, Xihai Xu, Lingling Xia, Tingting Bian, Yanyan Liu, Hui Zhang, Xuejiao Ma, Huafa Yin, Qiulin Sun, Yufeng Gao, Ying Ye, Jiabin Li

**Affiliations:** ^1^ Department of Infectious Diseases, The First Affiliated Hospital of Anhui Medical University, Hefei, China; ^2^ Department of Infectious Diseases, Chaohu Hospital of Anhui Medical University, Hefei, China

**Keywords:** novel phlebovirus, severe fever with thrombocytopenia syndrome, invasive pulmonary aspergillosis, immunity, risk factors

## Abstract

Aspergillus-related disease was confirmed to be associated with immune disorders in patients, severe patients with severe fever with thrombocytopenia syndrome (SFTS) infected by novel phlebovirus were confirmed to have severe immune damage including cellular immunosuppression and cytokine storms. Secondary invasive pulmonary aspergillosis (IPA) in severe SFTS patients can increase fatality rate. This study investigated early-warning predictive factors of secondary IPA in severe SFTS patients. Receiver operating characteristic analysis was used to assess the value of immune parameters to predict IPA in SFTS patients. The cut-off values of CD4^+^ and CD8^+^ T-cell counts to predict IPA were 68 and 111 cells/mm^3^, with sensitivities of 82.6% and 72%, and specificities of 56.7% and 83.3%, respectively. Cut-off values of IL-6, TNF-α, IL-8, and IL-10 to predict IPA incidence in critically ill SFTS patients were 99 pg/mL, 63 pg/mL, 120 pg/mL, and 111 pg/mL, with sensitivities of 90.0%, 86.7%, 83.3% and 90.0% and specificities of 80.4%, 71.7%, 82.6% and 65.2%, respectively. Lower CD4^+^ and CD8^+^ T-cells counts, higher levels of IL-6, TNF-α, IL-8 and IL-10, higher incidence of pancreatic and renal damage, early antibacterial therapy of carbapenems, and intensive care unit admission were risk factors of IPA in SFTS patients. Multivariate logistic regression analysis indicated counts of CD4^+^ T-cells <68 cells/mm^3^ combined with CD8^+^ T-cells <111 cells/mm^3^ (odds ratio [OR] 0.218, 95% confidence interval [CI] 0.059–0.803, *p*=0.022), IL-6 >99 pg/ml combined with IL-10 >111 pg/ml (OR 17.614, 95% CI 2.319–133.769, *p*=0.006), and brain natriuretic peptide level >500 pg/ml (OR 13.681, 95% CI 1.994–93.871, *p*=0.008) were independent risk factors for IPA in SFTS patients. The mortality in the IPA group was significantly higher than in the non-IPA group (*p*=0.001). Early antifungal treatment of IPA patients was significantly associated with improved survival (log-rank, *p*=0.022). Early diagnosis of IPA and antifungal treatment can improve the prognosis of SFTS patients. Besides, we speculate SFTS may be as a host factor for IPA.

## Introduction

Severe fever with thrombocytopenia syndrome (SFTS) is caused by a novel phlebovirus in the Bunyaviridae family named SFTS virus (SFTSV) (1). Contact with ticks appears to be a major risk factor for acquiring SFTSV, SFTSV could also be transmitted from person to person *via* infected blood (2). SFTS has been becoming an increasing public health threat in East Asia due to high morbidity and mortality (3–9). Secondary infection has contribution to the fatality of virus infection. Invasive pulmonary aspergillosis (IPA) have been reported to improve mortality in SFTS patients (10, 11). However, why severe SFTS patients are at risk for invasive pulmonary aspergillosis is not yet clear. Secondary infection as IPA should be concerned in severe SFTS patients.

Aspergillus-related disease associated with immune disorders in critically ill patients has been reported (12). Severe SFTS patients were confirmed to have immune cell dysfunction, and humoral immune system dysregulation which may predispose for IPA (13–16), however, measurable real-time indicators of predictor factors for IPA in severe SFTS patients are lack. Diagnosis and appropriate antifungal therapy of IPA are often delayed due to lack of factors to evaluate IPA in severe SFTS patients. This study was to investigated the early-warning predictors for IPA occurrence in severe SFTS patients to reduce mortality.

## Methods

### Study Population

SFTS patients with abnormal findings by CT scan of the lungs were included in the study at the First Affiliated Hospital of Anhui Medical University from March 2015 to December 2019. Patients with chronic lung disease, chronic kidney disease, and chronic liver disease were excluded. All SFTS patients were diagnosed with SFTSV RNA-positive *via* blood samples. SFTSV was amplified from serum samples using specific primers and probes by real-time reverse-transcription polymerase chain reaction (RT-PCR) under the conditions previously described (17). The demographic data, clinical manifestations, antibacterial therapy, antifungal therapy, and laboratory test results of patients were analyzed. Severely ill patients were diagnosed based on the guidelines for the prevention and treatment of fever with thrombocytopenia syndrome (2010 Edition) (18). Written informed consent was provided by all patients following the Declaration of Helsinki. The study and relevant experiments were approved by the local Ethics Committee of Anhui Medical University.

### Laboratory Tests

The functions of the liver, kidney, and pancreas, and myocardial enzymes were detected by routine biochemistry tests in a hospital laboratory after patient admission. Laboratory tests including routine blood test, B-type natriuretic peptide (BNP), C-reactive protein (CRP), procalcitonin (PCT), galactomannan (GM), and (1, 3)-β-D-glucan (G) were detected by routine tests after patient admission.

As described in our previous study (13), inflammatory mediators including interleukin (IL)-6, IL-10, IL-8, and tumor necrosis factor (TNF)-α were measured in plasma samples from patients using MILLIPLEX MAP human cytokine/chemokine magnetic bead panel kits (Merck Millipore, Germany) according to the manufacturer’s instructions (Luminex 200 System, Life Technologies, Grand Island, NY, USA).

Peripheral EDTA blood was stained with the following mouse anti-human monoclonal antibodies (Beckman Coulter Immunotech SAS, Marseille, France) according to the manufacturer’s recommendations: CD45-Krome orange (KO), CD3-fluorescein isothiocyanate (FITC), CD4-phycoerythrin (PE), CD8-allophycocyanin (APC), and fluorospheres. White blood cells were washed and resuspended in phosphate-buffered saline (PBS), then analyzed for CD4^+^ and CD8^+^ T lymphocytes on a Beckman Coulter Navios flow cytometer (Beckman Coulter, Miami, FL, USA).

### Definition of Invasive Aspergillosis Among SFTS Patients

Based on the 2019 EORTC-MSG consensus definitions (19), IPA was diagnosed based on clinical, radiological, and mycological criteria as the presence of the following criteria: 1) signs and symptoms of IPA such as cough, expectoration, and abnormal findings by CT scan of the lungs; 2) mycological criteria: histopathologic evidence of hyphae with recovery of Aspergillus from tissue, *Aspergillus fumigatus* recovered by culture from sputum, broncho alveolar lavage (BAL), or bronchial brush, positive GM as any 1 of the following: single serum or plasma: ≥1.0, BAL fluid: ≥1.0, single serum or plasma: ≥0.7 and BAL fluid ≥0.8. Severe neutropenia is defined as an absolute neutrophil count less than 500× 10^6^/L. All the SFTS patients included in this study were sorted into IPA and non-IPA groups. According to the EORTC/MSG diagnostic criteria, patients with proven and probable IPA were classified as the IPA group.

### Statistical Analysis

Data were analyzed using SPSS, version 20.0 (SPSS Inc., USA). Quantitative variables were expressed as means ± standard deviation (SD) or as medians (range). Univariate analysis was performed by utilizing the independent Student’s *t*-test or the Mann-Whitney *U*-test for between-group comparisons of continuous variables and chi-squared tests for between-group comparisons of qualitative data. Multiple logistic regression analysis was used for between-group comparisons of differences in clinical and biochemical variables. Survival analysis comparisons between groups were analyzed by the log-rank (Mantel-Cox) test using GraphPad Prism 5.0 (GraphPad Software, San Diego, USA). All significance tests were two-tailed, and differences were considered statistically significant at *p ≤* 0.05.

## Results

### Characterization of SFTS Patients

**Figure 1:**
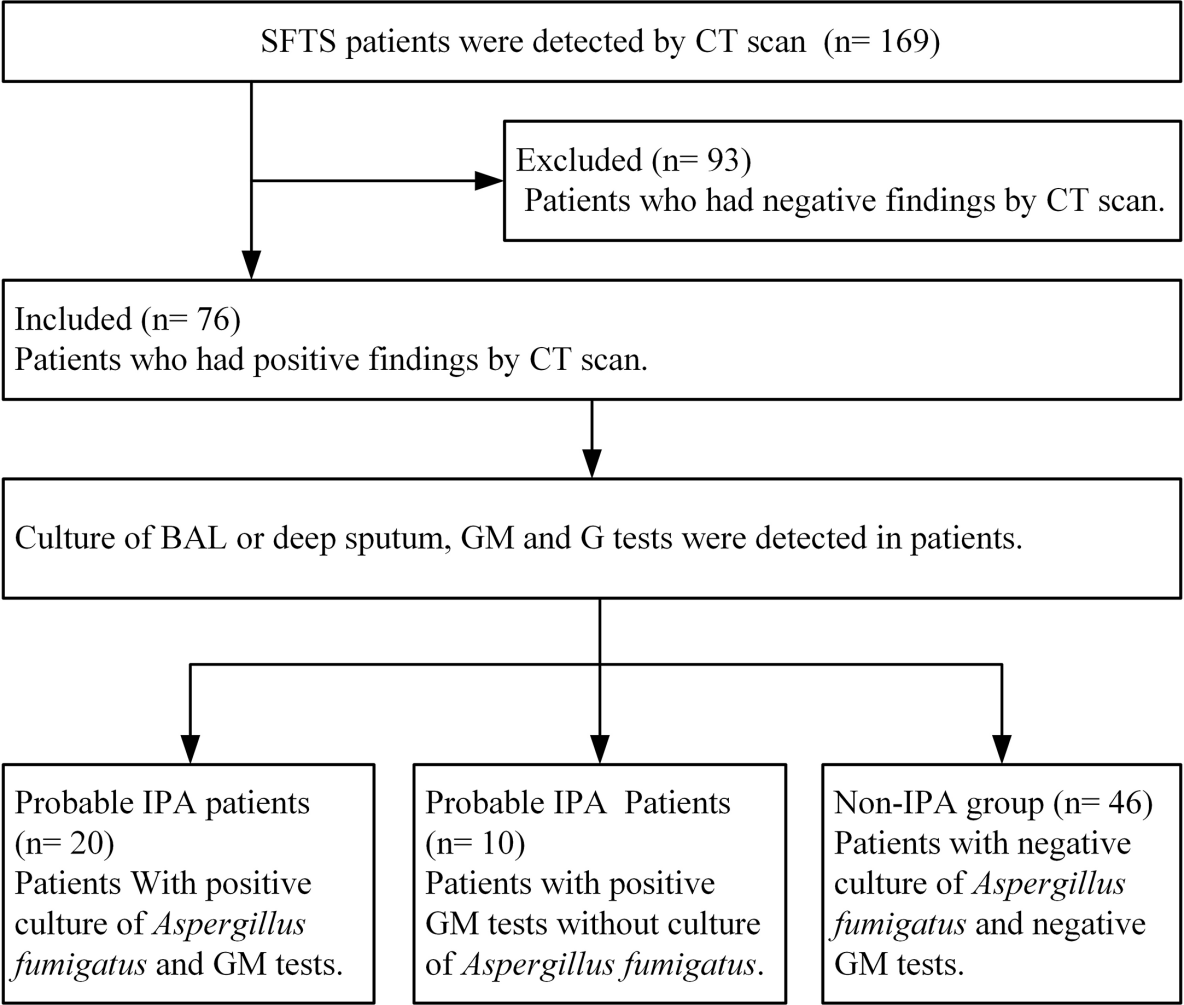
Diagram flow of severe patients with severe fever with thrombocytopenia syndrome included in this research. IPA, invasive pulmonary aspergillosis; SFTS, severe fever with thrombocytopenia syndrome; GM, galactomannan; G, (1,3)-β-D-glucan; BAL, broncho alveolar lavage.

Among 169 SFTS patients from March 2015 to December 2019, 76 severe SFTS patients who had abnormal findings by CT scan of the lungs with a new or progressive radiographic infiltrate were included to be studied. Among 76 SFTS patients, the mean age was 66.75 ± 9.55 years (47–87 years), 44 patients were male, 6 patients had diabetes mellitus, and all the patients were from rural areas. According to the 2019 EORTC-MSG diagnostic criteria, 30 (39.5%) of the 76 severe SFTS patients were diagnosed as IPA. As shown in **Figure 1**, twenty patients were diagnosed as probable IPA with positive culture of *Aspergillus fumigatus* in sputum or BAL, the GM levels were 0.95 ± 0.49 μg/L in serum among the 20 patients and 1.05 ± 0.25μg/L in BAL among 7 patients. Ten patients were diagnosed as probable IPA with positive GM (1.27 ± 0.23μg/L) in the serum.

All the 76 severe SFTS patients had fever with a mean duration of 10 ± 5 days, showed in **Table 1**, the severe patients had clinical symptoms and signs as cough, hemoptysis, dyspnea, gastrointestinal symptoms, and central nervous system symptoms. Thirty-five (46.1%) patients had severe neutropenia, 71 (93.4%) had multiple organ damage (liver damage 96.1%, pancreatitis 86.8%, kidney damage 50%, and myocardial damage 90.7%). Damage of the anticoagulant system was demonstrated by a prolonged anginal partial thromboplastin time (APTT) of 71 seconds (interquartile range 55-94 seconds).

**Table 1:** Comparisons of clinical and laboratory characteristics between IPA and non-IPA patients with severe fever with thrombocytopenia syndrome.

Index	All patients (N = 76)	IPA patients (N = 30)	Non-IPA patients (N = 46)	*P* value
Clinical characteristics, symptoms or signs				
Age, years	66.75 ± 9.55	66.53 ± 9.05	66.89 ± 9.87	0.874
Male, N (%)	46(60.5)	20(66.6)	26(56.5)	0.376
Duration of fever, (days)	10.55 ± 5.07	11.63 ± 5.76	9.85 ± 4.5	0.135
Cough, N (%)	48(63.1)	24(80)	24(52.2)	0.014
Hemoptysis, N (%)	14(18.4)	10(33.3)	4(8.7)	0.007
Dyspnea, N (%)	19(25)	12(40)	7(15.2)	0.015
CNS symptoms, N (%)	47(61.8)	26(86.6)	21(45.6)	0.000
Alimentary symptoms, N (%)	33(43.3)	13(43.3)	20(43.4)	0.924
Laboratory parameters				
Neutropenia, N (%)	35(46.1)	20(66.6)	15(32.6)	0.004
Platelet count (x10^9^/L)	33(20-48)	21(16-33)	38(28-53)	0.000
CD4^+^ T-cells, (cells/mm^3^, normal range 410–1590)	96(56-139)	58(44-97)	122(75-200)	0.000
CD8^+^ T-cells, (cells/mm^3^, normal range 190–1140)	111(61-168)	67(41-104)	142(99-218)	0.000
IL-6,(pg/mL, normal range 0–5.9)	95(35-197)	172(132-297)	44(25-94)	0.000
TNF-α, (pg/mL, normal range 0–8.1)	65(35-205)	137(73-232)	44(27-80)	0.000
IL-8, (pg/mL, normal range 0–62)	89(35-256.8)	223(126-365.5)	40(29-98)	0.000
IL-10, (pg/mL, normal range 0–9.1)	136(44-413.5)	260(135.7-509.2)	86(29-221)	0.000
BNP, (pg/mL, normal range 0–100)	174(75-512)	567(254-1016)	94(55-189)	0.000
G test, (pg/mL)	9.7(5-28.8)	19(9.8-56)	5(5-10)	0.000
GM test, (μg/L)	0.65 ± 0.45	1.06 ± 0.44	0.38 ± 0.17	0.000
PCT, (mg/L)	0.7(0.27-1.4)	0.49(0.18-1.52)	0.86(0.3-1.3)	0.127
CRP, (ng/mL)	15.1(6.5-43.1)	23.4(9-50.7)	10.8(3.3-35.3)	0.031
APTT, (seconds, normal range 28-42)	71(55-94)	89(58.9-104.4)	64.2(50-84)	0.005
MODS, N (%)	71(93.4)	30(100)	41(89.1)	0.062
Pancreatic damage, N (%)	66(86.8)	29(96.6)	37(80.4)	0.041
Renal damage, N (%)	38(50)	25(83.3)	13(28.3)	0.000
Myocardial damage, N (%)	69(90.8)	29(96.6)	40(86.9)	0.152
ICU admission, N (%)	7(9.2)	7(23.3)	0	0.001
DM, N (%)	6(7.9)	3(10)	3(6.5)	0.583
Carbapenems application, N (%)	28(36.8)	21(70)	7(15.2)	0.000
Death, N (%)	24(31.6)	16(53.3)	8(17.4)	0.001

Data are presented as the mean ± standard deviation, median (interquartile range), and proportion.

IL-6, interleukin-6; TNF-α, tumor necrosis factor-alpha; IL-8, interleukin-8; IL-10, interleukin-10; MODS, multiple organ dysfunction syndromes; ICU, intensive care unit; DM, diabetes mellitus. BNP, brain natriuretic peptide; G, (1,3)-β-D-glucan; CRP, C-reactive protein; PCT, procalcitonin; GM, galactomannan.

All severe SFTS patients had immune damage in this study. the CD4^+^ and CD8^+^ T lymphocytes were reduced obviously. Among the 76 severe patients, the median levels of CD4^+^ and CD8^+^ T lymphocytes were 96(interquartile range, 56-139) and 111(interquartile range, 61-168) cells/mm^3^, respectively. The proinflammatory cytokines IL-6 and TNF-α, and anti-inflammatory mediator IL-10 and IL-8 were upregulated. The median levels of IL-6, TNF-α, IL-10 and IL-8 were 95(interquartile range, 35-197), 65(interquartile range, 35-205), 136(interquartile range, 44-413.5) and 89(interquartile range, 35-256.8) pg/mL, respectively.

### Risk factors for IPA in SFTS patients

Among the 76 severe SFTS patients, 24(31.5%) patients including 16 in the IPA group and 8 in the non-IPA group died. The mortality rate in the IPA group was significantly higher than that in the non-IPA group (53.3% *vs* 17.4%, *p*=0.001).

A comparison of patients (**Table 1**) indicated the incidence of cough (80.0% *vs* 52.2%, *p*=0.014), hemoptysis (33.3% *vs* 8.7%, *p*=0.007), dyspnea (40.0% *vs* 15.2%, *p*=0.015), CNS symptoms (86.6% *vs* 45.6%, *p*=0.000), and neutropenia (66.6% *vs* 32.6%, *p*=0.004) were significantly higher in patients with IPA than in patients without IPA. Thirty patients with IPA had lower levels of CD4^+^ and CD8^+^ lymphocytes and platelets (all *p*=0.000), higher levels of IL-6, TNF-α, IL-8 and IL-10 (all *p*=0.000), higher levels of G, GM, CRP, and APTT (all *p*<0.05), and a higher incidence of pancreatic and renal damage (*p*<0.05) compared with non-IPA patients. No significant differences in underlying disease such as DM were found between IPA and non-IPA patients.

**Figure 2:**
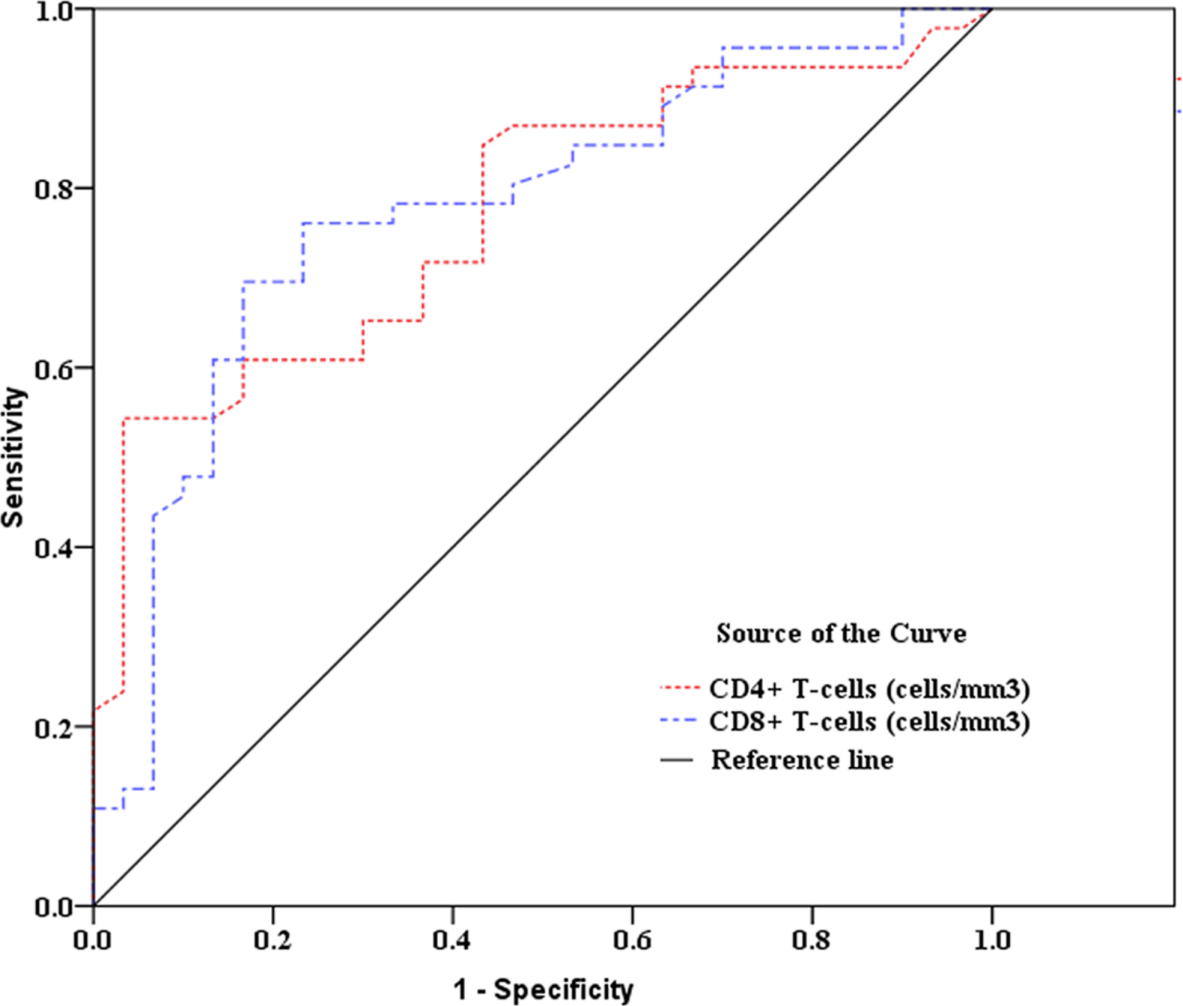
Receiver operating characteristic curve analysis of CD4^+^ and CD8^+^ T-cell counts to predict invasive pulmonary aspergillosis in severe patients with severe fever with thrombocytopenia syndrome.

**Figure 3:**
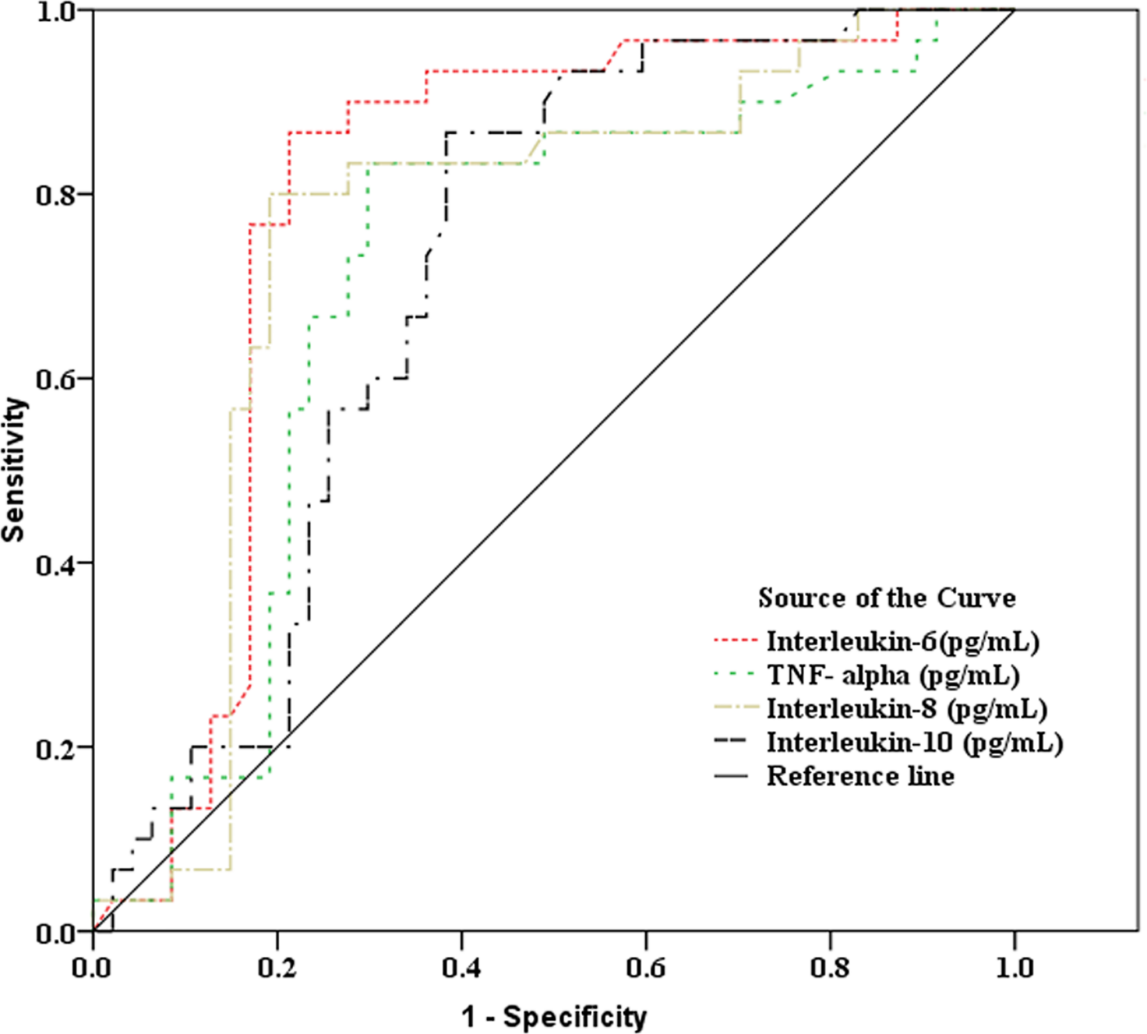
Receiver operating characteristic curve analysis of inflammatory mediators to predict invasive pulmonary aspergillosis in severe patients with severe fever with thrombocytopenia syndrome.

As showed in **Table 2** and **Figures 2**, **3**, receiver operating characteristic (ROC) analysis was used to assess the value of immune parameters to predict IPA in SFTS patients. The cut-off values of CD4^+^ and CD8^+^ T-cell counts to predict IPA were 68 and 111 cells/mm^3^, with sensitivities of 82.6% and 72%, and specificities of 56.7% and 83.3%, respectively. Cut-off values of IL-6, TNF-α, IL-8, and IL-10 to predict IPA incidence in severe SFTS patients were 99 pg/mL, 63 pg/mL, 120 pg/mL, and 111 pg/mL (**Table 2** and **Figures 2**, **3**), with sensitivities of 90.0%, 86.7%, 83.3% and 90.0%, and specificities of 80.4%, 71.7%, 82.6% and 65.2%, respectively.

**Table 2:** Receiver operating characteristic curve analysis of immune parameters predicting invasive pulmonary aspergillosis insevere patients with severe fever with thrombocytopenia syndrome.

Index	Cut off value	AUC (95%CI)	*P* value	sensitivity	specificity
CD4^+^T-cells, cells/mm^3^	68	0.777(0.673-0.881)	0.000	82.6	56.7
CD8^+^T-cells, cells/mm^3^	111	0.772(0.662-0.881)	0.000	72.0	83.3
IL-6, pg/ml	99	0.843(0.750-0.937)	0.000	90.0	80.4
TNF-α, pg/ml	63	0.755(0.641-0.869)	0.000	86.7	71.7
IL-8, pg/ml	120	0.797(0.690-0.905)	0.000	83.3	82.6
IL-10, pg/ml	111	0.752(0.644-0.860)	0.000	90.0	65.2

AUC, area under the curve; CI, confidence interval; IL-6, interleukin-6; TNF-α, tumor necrosis factor-alpha; IL-8, interleukin-8; IL-10, interleukin-10.

Because all SFTS patients were diagnosed with pulmonary infection, antibiotic treatment as carbapenems were used in the early stage of disease in 28 patients. Among the 28 patients, 21 developed IPA. Early antibacterial therapy of carbapenems was associated with higher incidence of IPA in SFTS patients (70% *vs* 15.2%, *p*=0.000). Seven patients were admitted to the intensive care unit, and they all developed IPA during hospitalization, indicating intensive care unit admission was a risk factor for IPA. In this study, no patients used corticosteroids.

As shown in **Table 3**, multivariate logistic regression analysis indicated that CD4^+^ T-cells <68 cells/mm^3^ combined with CD8^+^ T-cells <111 cells/mm^3^ (OR 0.218, 95% CI 0.059–0.803, *p*=0.022), IL-6 >99 pg/mL combined with IL-10 >111 pg/mL (OR 17.614, 95% CI 2.319–133.769, *p*=0.006), and BNP level >500 pg/mL (OR 13.681, 95% CI 1.994–93.871, *p*=0.008) were independent risk factors for IPA in SFTS patients.

**Table 3:** Multivariate logistic regression analysis of factors predicting invasive pulmonary aspergillosis in severe patients with severe fever with thrombocytopenia syndrome.

Index	β	OR	95%CI	*P-v*alue
CD4^+^T-cells <68 cells/mm^3^ and CD8^+^T-cells <111 cells/mm^3^	-1.523	0.218	0.059-0.803	0.022
IL-6>99 pg/ml and IL-10>111 pg/ml	2.869	17.614	2.319-133.769	0.006
BNP>500 pg/ml	2.616	13.681	1.994-93.871	0.008

CI, confidence interval; OR, odds ratio; IL-6, interleukin-6; IL-8, interleukin-8; BNP, brain natriuretic peptide.

### Prognostic Factors for Patients With IPA

**Figure 4:**
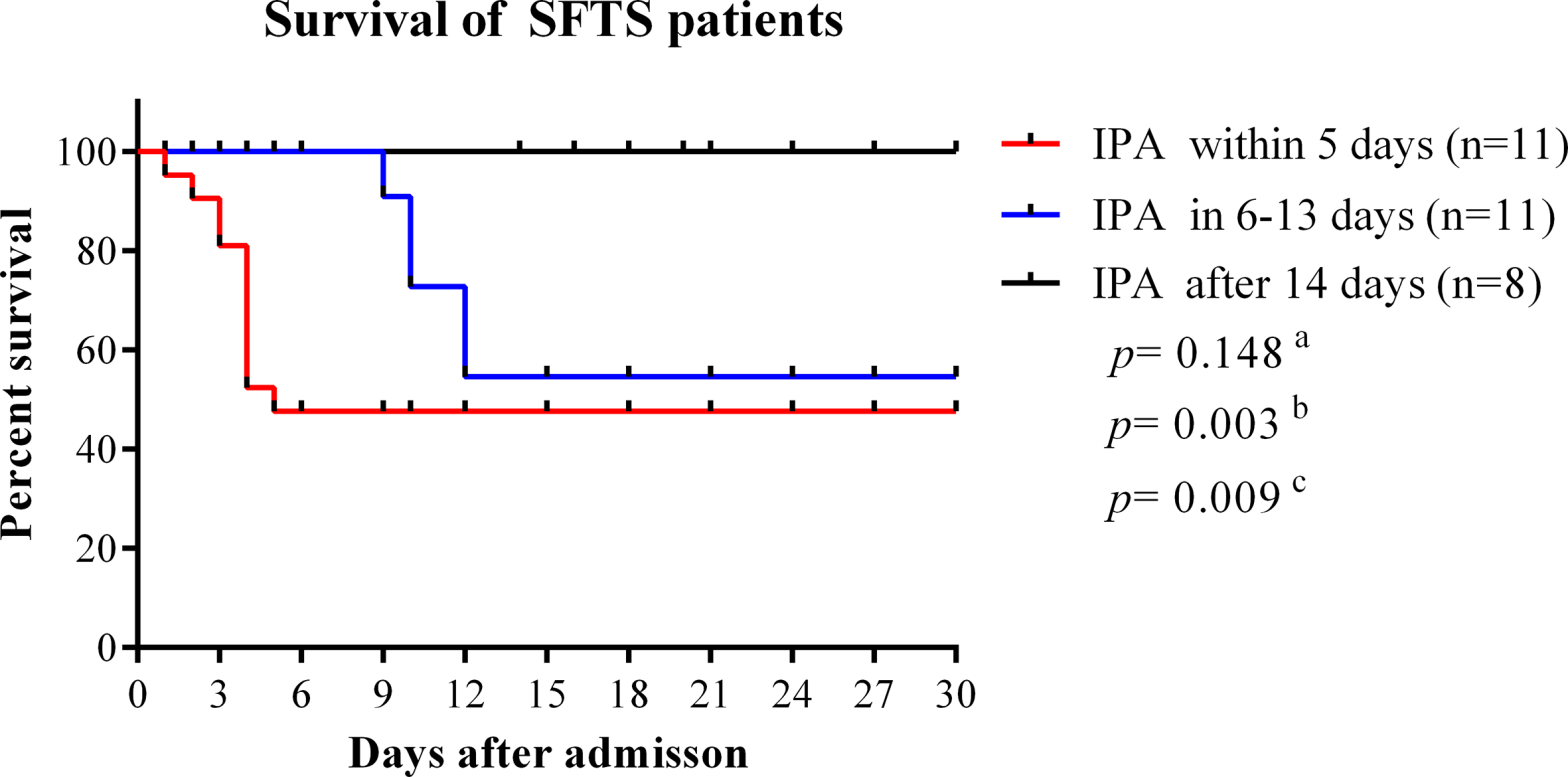
Survival curves were compared among SFTS patients with different time points of IPA occurrence. IPA, invasive pulmonary aspergillosis; SFTS, severe fever with thrombocytopenia syndrome. ^a^Comparison of survival curves between patients with IPA occurrence within 5 days and 6–13 days from disease onset. ^b^Comparison of survival curves between patients with IPA incidence within 5 days and after 14 days from disease onset. ^c^Comparison of survival curves between patients with IPA incidence 6–13 days after onset and 14 days after disease onset.

**Figure 5:**
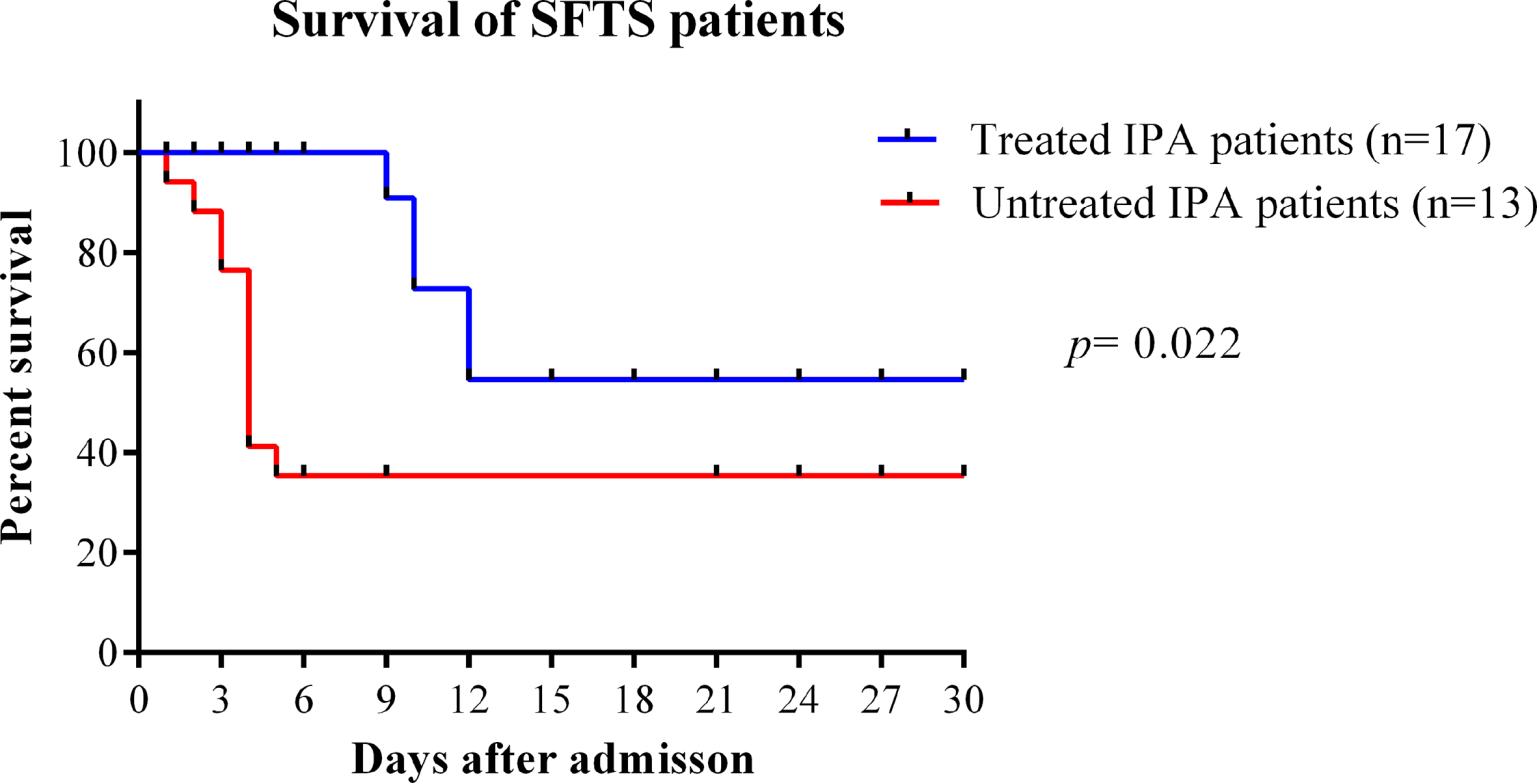
Survival curves were compared between SFTS patients with IPA treated with antifungal treatment or untreated. IPA, invasive pulmonary aspergillosis; SFTS, severe fever with thrombocytopenia syndrome.

In our study, the median time from disease onset to IPA diagnosis was 10 days (interquartile range, 8–14). The median time from admission time to IPA diagnosis was 5 days (interquartile range, 3–9). As shown in **Figure 4**, the case fatality rate was significantly reduced in patients with IPA occurrence 14 days after onset compared with IPA occurrence 6–13 days after onset (log-rank, *p*=0.0001). The earlier occurrence of IPA was associated with a higher fatality rate. Early antifungal treatment against IPA was significantly associated with improved survival (log-rank, *p*=0.022) in severe SFTS patients (**Figure 5**).

## Discussion

Non-neutropenic critically ill patients with COPD, cirrhosis, severe influenza and neutropenic critically ill patients have been described in association with invasive aspergillosis (20–22). SFTSV has the ability to attenuate cellular and humoral immune responses of host, the dysregulated immune system as cellular immunosuppression and immune paralysis may contribute to the development of IPA in severe SFTS patients. In this study, severe SFTS patients with secondary IPA were studied.

IPA contributed to increase mortality of SFTS patients. Clinically, when critically ill patients suffered from secondary infection, bacterial infection was considered firstly, followed by fungal infection, and therefore antibacterial treatment was emphasized. However, we found critically ill SFTS patients can be infected with aspergillus in the early-stage of disease in this study. Besides, the occurrence of IPA at the early-stage of disease course was identified to be associated with a higher mortality rate. What should be noted is that, all our patients were from rural areas where the tick-borne was epidemic, the living characteristics of SFTS patients indicated they were easily to be exposed to aspergillus in the environment in daily life. Under severely ill condition caused by SFTSV, they would be more prone to aspergillus infection. Besides, the pathogenic characteristics of cellular immunosuppression, humoral immune regulation disorders, severe neutropenia and MODS in SFTS patients may increase susceptibility to IPA. Early diagnosis and effective antifungal treatment against IPA have important value to improve the prognosis of SFTS patients. Therefore, identifying predisposing factors of IPA in severe SFTS patients as an early warning of IPA is very important. According to our results, agranulocytosis, attenuation of CD4^+^ and CD8^+^ T-cell, humoral immune imbalance, pancreatic and renal damage can predict the occurrence of IPA.

In this research, CD4^+^ and CD8^+^ T-cell counts were confirmed to be useful parameters to early prediction of IPA in SFTS patients with high sensitivities and specificities of cut-off values. T lymphocyte cells in SFTS patients decreased seriously by SFTSV invasion in severe SFTS patients. We found lower counts of CD4^+^ and CD8^+^ T-cells were independent predictors for a high risk of IPA in severe SFTS patients. Previous study has showed that fungal infections were associated with impaired cell-mediated immunity and CD4^+^ T-cell responses were critical for protection against invasive fungal infections (IFIs) (23). CD8^+^ T-cells were also reported to be independent predictors for a high risk of IPA and early mortality in critically ill immunocompromised patients with IPA (12). Our research showed that CD4^+^ T-cell counts <68 cells/mm^3^ combined with CD8^+^ T-cell counts <111 cells/mm^3^ were independent risk factors for IPA in critically ill SFTS patients. To the best of our knowledge, this is the first report to evaluate the important early warning role of CD4^+^ and CD8^+^ T-cell counts for IPA in critically ill SFTS patients.

Our study confirmed the early-warning value of inflammatory mediators to predict IPA in severe SFTS patients with high sensitivities and specificities of cut-off values. The inflammatory response in SFTS patients was confirmed to play an important role in the pathogenesis of SFTSV (13, 14). In the acute-stage in severe SFTS patients, many pro- and anti-inflammatory mediators are produced, resulting in systemic inflammatory response syndrome (SIRS) and compensatory anti-inflammatory response syndrome (CARS). SIRS lead to cell death and organ dysfunction, CARS lead to immunosuppression which increases susceptibility to secondary infection (24, 25). When SIRS and CARS coexist, a more serious inflammatory disorder will occur, termed mixed antagonistic response syndrome, which increases the occurrence of secondary infection. Our previous research has revealed cytokine storm with high levels of pro- and anti-inflammatory cytokines in severe SFTS patients (13). Cytokine storms have been reported as a major pathophysiologic mechanism that aggravates leukopenia and thrombocytopenia (26). Leukopenia is a well-known risk factor for IPA, platelets have the ability to block *Aspergillus fumigatus* germination and hyphal elongation (27). All these characteristics in severe SFTS can make patients more vulnerable to IPA. Pro-inflammatory cytokine such as IL-6, and anti-inflammatory cytokine such as IL-10, have key roles in maintaining the immune balance. Overexpression of IL-10 has been reported to be a risk factor for invasive aspergillosis in patients after stem-cell transplantation (28). Measurement of immunocompetence would be possible to detect SFTS patients who are at high risk of developing IPA at an earlier time. In this study, we’ve got quantitative indicators of inflammatory factors to predict IPA, IL-6 >99 pg/mL combined with IL-10 >111 pg/mL in SFTS patients was confirmed to be an independent predictor for the high risk of IPA.

BNP are known to reflect cardiac damage and circulation overload, it was also reported to be independently associated with risk of infection including pneumonia, urinary tract infections, bloodstream infections, and cellulitis (29). In addition, the results in this research concluded that BNP was an important independent predictor for IPA occurrence in severe SFTS patients. Clinically, monitoring BNP in critical SFTS patients is of great significance for disease condition evaluation and the early diagnosis of IPA.

The course of SFTS patients can be divided into the initial stage of fever, acute stage, and recovery period or death. According to our results, patients with IPA occurrence within 2 weeks from disease onset had a significantly higher mortality rate than those with IPA occurrence after 2 weeks from onset, suggesting the risk of death from IPA is higher in the acute phase. Within the first 2 weeks of the disease, severe SFTS patients have impaired immune system and MODS. Occurrence of IPA within 2 weeks would be fatal for severe SFTS patients. Our results revealed that early antifungal therapy significantly reduced mortality in SFTS patients with IPA, similar to a previous report (11). However, in clinical practice, early diagnosis and antifungal therapy of IPA represents a challenge due to lack of diagnostic algorithm. SFTS patients who are considered at risk for IPA, should raise clinician awareness.

In conclusion, severe SFTS patients have more risk factors to develop IPA in the early stage of disease, which can increase mortality. CD4^+^ and CD8^+^ T-cell counts, IL-6 and IL-10 levels, and BNP level have important predictive values for the early diagnosis of IPA, which in combination with appropriate antifungal treatment can contribute to a better prognosis for severe SFTS patients. Moreover, we speculate SFTS may be as a host factor for IPA. Our results will be of great value to the field of fungal infections and optimal practices for diagnosis and treatment of SFTS disease.

## Data Availability Statement

The original contributions presented in the study are included in the article/supplementary material. Further inquiries can be directed to the corresponding author.

## Ethics Statement

This study was approved by the local Ethics Committee of the First Affiliated Hospital of Anhui Medical University. The patients/participants provided their written informed consent to participate in this study.

## Author Contributions

LH and JL conceived and designed the project, and composed the manuscript. QK and CY collected clinical data and analyzed the data. YYL, HZ, and TB performed the laboratory experiments. QK, CY, and XM performed the flow cytometry data analyses. LH, XX, LX, YY, HY, QS, YG, and JL gave diagnosis and treatment of the patients. All authors contributed to the article and approved the submitted version.

## Funding

This study was supported by the National Natural Science Foundation of China (No. 81172737 and No. 81101313).

## Conflict of Interest

The authors declare that the research was conducted in the absence of any commercial or financial relationships that could be construed as a potential conflict of interest.
